# The evaluation of a tailored intervention to improve the management of suspected viral encephalitis: protocol for a cluster randomised controlled trial

**DOI:** 10.1186/s13012-014-0201-1

**Published:** 2015-01-27

**Authors:** Ruth Backman, Robbie Foy, Peter J Diggle, Rachel Kneen, Sylviane Defres, Benedict Daniel Michael, Antonieta Medina-Lara, Tom Solomon

**Affiliations:** Department of Clinical Infection, Microbiology and Immunology, Institute of Infection and Global Health, University of Liverpool, Ronald Ross Building, 8 West Derby Street, Liverpool, L69 7BE United Kingdom; Leeds Institute of Health Sciences, University of Leeds, Charles Thackrah Building, 101 Clarendon Road, Leeds, LS2 9LJ United Kingdom; Department Epidemiology and Population Health, Institute of Infection and Global Health, University of Liverpool, Ronald Ross Building, 8 West Derby Street, Liverpool, L69 7BE United Kingdom; Department of Neurology, Alder Hey Children’s NHS Foundation Trust, Eaton Road, Liverpool, L12 2AP United Kingdom; Royal Liverpool and Broadgreen University Hospitals Trust, Liverpool, L7 8XP United Kingdom; The Walton Centre NHS Foundation Trust, Lower Lane, Fazakerly, Liverpool, L9 7LJ United Kingdom; Health Economics Group, University of Exeter Medical School, Veysey Building, Salmon Pool Lane, Exeter, EX2 4SG United Kingdom

**Keywords:** Encephalitis, Cluster randomised controlled trial, Guideline implementation, Protocol

## Abstract

**Background:**

Viral encephalitis is a devastating condition for which delayed treatment is associated with increased morbidity and mortality. Clinical audits indicate substantial scope for improved detection and treatment. Improvement strategies should ideally be tailored according to identified needs and barriers to change. The aim of the study is to evaluate the effectiveness and cost-effectiveness of a tailored intervention to improve the secondary care management of suspected encephalitis.

**Methods/Design:**

The study is a two-arm cluster randomised controlled trial with allocation by postgraduate deanery. Participants were identified from 24 hospitals nested within 12 postgraduate deaneries in the United Kingdom (UK). We developed a multifaceted intervention package including core and flexible components with embedded behaviour change techniques selected on the basis of identified needs and barriers to change. The primary outcome will be a composite of the proportion of patients with suspected encephalitis receiving timely and appropriate diagnostic lumbar puncture within 12 h of hospital admission and aciclovir treatment within 6 h. We will gather outcome data pre-intervention and up to 12 months post-intervention from patient records. Statistical analysis at the cluster level will be blind to allocation. An economic evaluation will estimate intervention cost-effectiveness from the health service perspective.

**Trial registration:**

Controlled Trials: ISRCTN06886935.

**Electronic supplementary material:**

The online version of this article (doi:10.1186/s13012-014-0201-1) contains supplementary material, which is available to authorized users.

## Background

There is evidence that the current clinical management of serious acute neurological infections is suboptimal [1-3]. Encephalitis, inflammation of the brain tissue, is most commonly caused by herpes simplex virus in the United Kingdom (UK) [4,5]. When herpes simplex virus encephalitis is treated promptly with aciclovir, there is a significant improvement in patient outcomes [6,7]. Encephalitis affects between five and eight people per 100,000 per year [8]. Sequelae after hospital discharge can include significant morbidities such as epilepsy, memory loss, and speech and behavioural disorders [9,10], which also impair patients return to work [10].

Whilst herpes simplex virus encephalitis is relatively rare [5], clinical presentations including features consistent with suspected encephalitis occur relatively frequently but in different ways to other brain injuries. Encephalitis typically presents with one or more of headache, fever, new-onset seizures, altered consciousness, and behavioural disturbances [11]. This variable and non-specific presentation often results in delayed diagnosis, especially in children who may only present with fever and irritability [12]. Furthermore, delays in using the main diagnostic technique, lumbar puncture, may further delay treatment [13-16].

Clinical guidelines have been developed in response to these problems [1-3,5,17]. However, simple dissemination of clinical guidelines is often unlikely to bring about significant changes in clinical practice [18-20]. Furthermore, interventions to implement clinical guidelines should ideally be based upon a diagnosis of barriers to change, preferably focusing on those most amenable to change [21].

### Aims

We developed a multifaceted intervention package including core and flexible components with embedded behaviour change techniques selected on the basis of identified needs and barriers to change (Backman, submitted). We will evaluate the effectiveness and cost-effectiveness of a tailored intervention to improve the secondary care management of suspected encephalitis.

## Methods

### Study design

Participating sites will be randomly allocated to intervention or control (no intervention) arms in a cluster randomised controlled trial.

### Participants

#### Hospitals

This trial takes place in the context of ENCEPH UK—Understanding and Improving the Outcome of Encephalitis, an ongoing research programme assessing the epidemiology and clinical outcomes of encephalitis. In order to reduce the likelihood of any unintended co-intervention effects we sought hospitals not directly participating in other ENCEPH UK studies. Sites had to have facilities to perform lumbar punctures and neuroimaging and willing to be randomised to intervention or control arms. We aimed to recruit a range of types of hospital, providing secondary, tertiary, (specialist) and paediatric care, to broadly represent national provision and improve the generalizability of subsequent findings.

We were aware that trainee doctors, one key target intervention group, work and rotate between different hospitals within postgraduate deaneries. If we randomised hospitals to intervention and control arms within the same deanery, there would be a risk of contamination. We therefore used deaneries as the unit of randomisation to minimise contamination.

**Figure 1:**
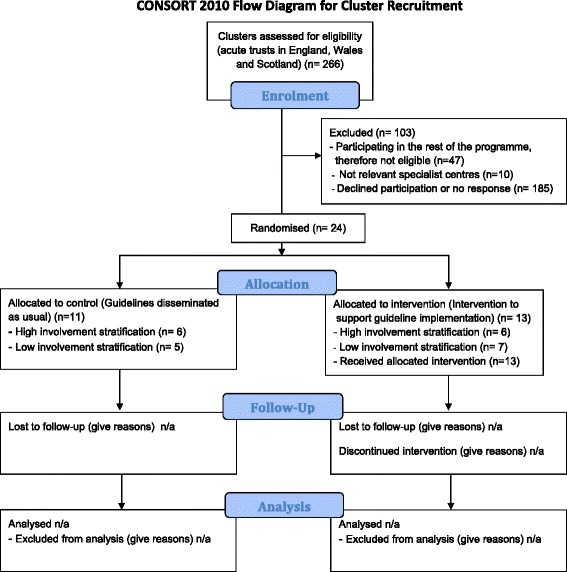
**CONSORT 2010 checklist of information to include when reporting a cluster randomised trial.**

We assessed all 266 acute trusts in England, Wales and Scotland for eligibility (Figure 1 and Additional file 1 detail a full CONSORT checklist). After excluding 47 participating in other ENCEPH UK studies and 10 specialist hospitals not usually providing routine care for suspected encephalitis patients, e.g. orthopaedic hospitals, we invited 209 hospitals to participate via senior medical members of staff.

#### Patients

We will identify records of patients with features suggestive of suspected encephalitis using three sets of criteria adapted from previous studies [1,22].

#### Method 1

Mandatory

- Acute or sub-acute (<4 weeks) alteration in consciousness, cognition, personality or behaviour persisting for more than 24 h. Personality/behaviour change includes: agitation, psychosis, somnolence, insomnia, catatonia, mood liability, altered sleep pattern and (in children) new-onset enuresis or irritability.

Plus any two of:

- Fever (≥38°C) or prodromal illness—acute or sub-acute
- New-onset seizures
- Focal neurological signs of acute or sub-acute onset, including focal weakness, oromotor dysfunction, movement disorders (chorea, athetosis, dystonia, hemiballismus, stereotypies, orolingual dyskinesia and tics) including Parkinsonism (bradykinesia, tremor, rigidity and postural instability) and amnesia
- Pleocytosis: cerebrospinal fluid white cell count of more than four cells per microlitre
- Neuroimaging compatible with encephalitis
- Electroencephalogram (EEG) compatible with encephalitis

#### Method 2

There was an initial clinical suspicion of encephalitis.

#### Method 3

There was a clinical suspicion of encephalitis, and the patient died before investigations were completed.

### Intervention package

Using theoretically informed semi-structured interviews based upon the Theoretical Domains Framework [23,24], we explored barriers and enablers to diagnosing and managing patients with suspected encephalitis, specifically performing lumbar punctures and initiating antiviral therapy within 6 h (Backman, submitted). We mapped identified barriers and enablers to the patient pathway. We matched behaviour change techniques targeting clinicians to the most salient barriers and enablers and embedded them within an intervention package [25].

The intervention package comprised ‘core’ interventions and, to allow for local flexibility, ‘optional’ interventions (Table 1). We defined ‘core’ interventions as those which we anticipated all hospitals being able to use. These included educational and action planning meetings, feedback of pre-intervention audit data and provision of lumbar puncture kits within refillable boxes. We defined ‘optional’ interventions which hospitals could use depending upon local resources and skills. These included decision support via phone apps and algorithms, an online quiz, prompts and posters, personalised invitation letters to attend educational meetings and a quality improvement cycle pack. Table 1 gives full details of the package following the Template for Intervention Description and Replication (TIDieR) reporting guidance [26].

**Table 1:** **Intervention description using the TIDieR guidelines for intervention reporting** [26]

**Intervention component**	**Overview of component**	**Materials**	**Who provided**	**Modes and frequency of delivery**	**Where it will take place**	**Tailoring**
Training day (core component)	Investigators were invited to attend a training day where the intervention was showcased and key behaviour change techniques to be communicated to their trainees were highlighted	Senior clinicians were provided with all the paper-based intervention materials in a bound form for reference. Materials were also provided in an electronic form for all study team members	Key behaviour changes and materials were delivered by the study team and a representative from The Encephalitis Society also shared their patient journey	Delivered once per site at the start of the intervention	Took place in a central location with additional local meetings as required	Feedback from this session led to the modification of intervention materials to make them more applicable to each hospital
Action planning meeting (core component)	Following training, investigators were asked to plan an implementation of intervention components	A form was provided which provided key topics to discuss and plan around	Each local PI ran the meeting	Training preceded this meeting, and it was requested that at least one was held prior to any educational sessions	Within each hospital with core team members	Sites could meet as frequently as required
Audit and feedback newsletter (core component)	An audit and feedback newsletter was produced with personalised data alongside an action planning element	Electronic and laminated copies were provided which contained personalised audit data, a link to the guidelines to promote action planning, and space to add local clinical leaders to add credibility	The study team provided the materials with the local clinical leaders distributing to their team	This was delivered electronically and in hard copy for distribution and display alongside all other intervention components	This will be displayed within the hospital nearby the other components such as the poster or the guideline algorithm	This newsletter was personalised to contain audit data from each hospital. It also contained a comparison with other anonymised hospitals, as well as to the recommended time frames for care
Lumbar puncture box (core component)	A refillable box with all the key equipment to perform a lumbar puncture was provided with sample collection information which could be locally modified as required	A box containing equipment for the procedure alongside a sheet detailing sample collection was provided. Adult and paediatric boxes were available due to different sample collection requirements	The study team supplied 2–6 boxes to each hospital as required	Boxes were delivered at the start of the intervention period and on an *ad hoc* basis	Boxes were placed in relevant locations as designated by each hospital	The sample sheet could be locally modified by each site to accommodate sample procedures
	Pre-made lectures with integrated behaviour change techniques were produced for the following uses: - A session focused upon the diagnostic lumbar puncture - A session focused upon the management of suspected encephalitis - A session for nurses on how to help with lumbar punctures	Pre-made lectures were provided alongside a range of other multimedia resources including: - A DVD showing nurses how to assist with the procedure - Two clinical vignettes - The Encephalitis Society YouTube channel - TS’ ‘Big Brain’ event on YouTube	The study team provided the resources for the local team to deliver as required	Sites were able to choose the frequency of delivery, with a recommended minimum of one per 6 months	All training will take place within the hospital and will be delivered by clinicians	These materials can be locally modified with a core set of slides so preserve behaviour change integrity. Furthermore, these are all modified for use in both an adult and paediatric setting and can be used as often as required by the local team
Educational survey (optional component)	An online multiple choice educational survey was developed with tailored questions for doctors and nurses. This online tool can be accessed at any time and all participants can download a certificate of completion	An online multiple choice educational survey was developed. A certificate of completion was awarded with additional checklists and action planning tools	Site PIs were able to circulate this online link to all junior doctors	The trainee would only complete the survey once with a certificate of completion	This could take place during a teaching session or during private study	Two surveys were available with questions tailored for doctors and nursing staff
ClickClinica [27] (optional component)	An app containing all current guidelines was developed. This has been promoted within our package both within the education and also within the personalised invitation letter as a useful tool	An app has been developed for use with iPhones whereby all guidelines are available in one place	This app was promoted through local clinical leaders	The link to download the app was within the educational sessions and contained within the direct mail letter	Guidelines can be checked and downloaded on the ward or within private study	None available for this component
Encephalitis Society leaflets and video (optional component)	The Encephalitis Society YouTube channel was included as a resource which could be incorporated into the education. Furthermore, patient leaflets will be disseminated to the investigators during the study	Printed materials for the patients and healthcare professionals were provided. These materials were also featured within the educational sessions	These were provided by The Encephalitis Society, forwarded by the study team and disseminated by the local clinical leaders	These were provided at the start and middle of the intervention period with reinforcement from the educational sessions	The YouTube videos could be used during the educational session or within a private study session as part of a critical reflection	None available for this component
	A quality improvement cycle (plan, do, study, act (PDSA)) was developed and included: - A summary page with the key guideline recommendations - A short list of key check box items to monitor current practice - An excel sheet which pre-plots the progress	Printed packs were provided alongside an electronic excel sheet with pre-plotting graphs to enable feedback	The study team provided all materials and these were locally disseminated by the clinical leader	Electronic materials were provided at the start of the study with printed materials provided within the 6-month update. Local clinical leaders were encouraged to ask a junior doctor to assess current management and feedback performance data	Within the ward where the local PI is based as part of a post-take ward round. The quality improvement graphs should also be displayed within this area	Junior doctors are able to feedback the areas of compliance they feel are most relevant to the department
Basis of modifiable care pathway (optional component)	The front sheet from the quality improvement cycle could also be modified to form the basis of a care pathway for suspected encephalitis patients. This will be locally driven and implemented at each site	A traffic light coloured sheet with the critical patient management items was provided as a basis to modify	The study team provided the resource and it was then adapted by the local clinical team	This was provided once at the start of the intervention	Local clinical leaders will modify and display in key areas of the hospital	This sheet was to be locally adapted if taken up
Algorithm (optional component)	The algorithm contained within the guidelines was reproduced with two additional features; a QR code which links directly to the guidelines and a box that contained details for local senior support	A laminated guideline algorithm was provided with a QR code to link to the original guidelines and a space for local modification with the additional of a local contact	The study team provided the materials, and they were disseminated throughout the hospital by the local clinical team	A minimum of five were provided to each site at the start and a further five were also provided after 6 months	These will be placed in key areas of the hospital as denoted by the local clinical leader	These are available in adult and paediatric forms and can be locally modified by the addition of a suitable contact if clinical decision support is required
Posters (optional component)	Posters with key symptoms and relevant QR codes were designed and graphics covered paediatric, adults and geriatrics	Three posters were provided to cover a variety of ages alongside the key clinical symptoms. A QR code to the guideline was also incorporated	The study team provided the materials, and they were disseminated throughout the hospital by the local clinical team	A minimum of five were provided to each site at the start, and a further five were also provided after 6 months	These will be placed in key areas of the hospital as denoted by the local clinical leader	None available for this component
Stickers (optional component)	Small stickers with ‘Think brain infection’ were produced for application to blood sample bottles	‘Think brain infection’ stickers were provided to raise awareness during the sample taking procedure	The local study team provided these on an *ad hoc* basis and the local clinical leaders affixed these to sample bottles as required	These were provided on an *ad hoc* basis with supplements sent within the 6-month update	These will be added to all relevant bottles as denoted by the study team	None available for this component
Invitation letter (optional component)	A template invitation letter from the consultant inviting the junior doctor to attend each of the education session was developed for local modification. Details of the lumbar puncture box and ClickClinica were also included	An electronic letter was made for each of the educational sessions	The study team provided the basis of a letter which will then be modified and sent by the consultant	This invitation letter can be sent prior to all educational sessions	Letters will be sent directly to the junior doctors to personally invite them to attend the educational session	The basis of the letter was provided for local modification

We presented the package to a 1-day meeting of senior doctors and nurses from intervention hospitals. We emphasised their roles in directly delivering the various intervention components locally and recommended that they each convene an action planning meeting on return to their hospitals.

### Outcomes

The primary outcome is a composite measure of the proportion of patients with suspected encephalitis whose care meets both of the following criteria: aciclovir given within 6 h from admission to hospital and a lumbar puncture performed within 12 h of hospital arrival unless clinically contraindicated.

Secondary outcomes comprise:

- The proportion of all adults started on intravenous aciclovir within an appropriate dosage range for a neurological presentation who met the definition of suspected encephalitis
- The proportion of all children started on intravenous aciclovir within an appropriate dosage range for a neurological presentation who met the definition of suspected encephalitis
- The proportion of patients with suspected encephalitis who had a lumbar puncture performed within 12 h unless there was a clinical contraindication
- The proportion of patients with suspected encephalitis who had a lumbar puncture at any point during the index presentation
- The proportion of patients with suspected encephalitis who had either magnetic resonance imaging (MRI) or computer tomography (CT) scan within 24 h of admission
- The proportion of patients with suspected encephalitis having had a lumbar puncture, who had the following cerebrospinal fluid (CSF) investigations performed: ratio of glucose within the cerebrospinal fluid and the serum calculated and having herpes simplex virus polymerase chain reaction (PCR) performed

We will also compare outcomes between adults and children.

### Data collection

We recognise that suspected cases would go through one or more of several types of hospital department but mainly paediatrics, neurology, infectious diseases, medical assessment unit, accident and emergency and microbiology. Staff in participating hospitals will identify suspected cases retrospectively by performing two mandatory searches, of discharge codes for encephalitis in the preceding 12 months and of all patients undergoing a lumbar puncture within the same time period. Hospitals can also find eligible patients via records of intravenous aciclovir prescriptions or of orders for cranial CT scan or MRI within the last 12 months. We are using these combined approaches to maximise likelihood of case identification and reduce differences in ascertainment between hospitals. We included both adult and paediatric cases.

Pre-intervention, we collected data to pilot outcome measures and provide data for the feedback intervention. We aimed for 30 adult and paediatric cases per hospital to allow for possible under-recruitment in some of the smaller hospitals; however, we later sought some additional cases from higher recruiting hospitals to better inform exploratory analyses of the baseline data and to compensate for lower recruiting sites. We plan to ask for a further 30 cases per hospital in the final trial data collection, thus the total patient sample size will be a maximum of 720. We anticipate a degree of imbalance between hospitals to achieve the total required sample size, limited by the upper limit of 40 cases per hospital.

Data are collected using structured case review forms. No patient identifiable information is sent to the central trial team. We trained data collectors, mainly nurses and trainee doctors, via face-to-face meetings and/or written briefing materials. We emphasised the need for a systematic approach to case identification to reduce the likelihood of selection bias. The trial research fellow (RB) is monitoring fidelity to the intervention via regular telephone and email contact with relevant hospitals. She is also collecting qualitative data from staff interviews and observational field notes in a sub-sample of four intervention hospitals data as part of a process evaluation.

### Sample size

Using pilot data from 315 patients across 26 hospitals in four deaneries, we estimated the standard deviations of the deanery and hospital random effects to be 0.244 and 1.108, respectively, and the current proportion of adherence to the primary outcome to be 5%. Table 2 shows the power of the likelihood ratio test for a significant difference between intervention and control arms as a function of *m*, the number of eligible patients recruited per hospital, and *p*, the proportion meeting the primary outcome criteria under the intervention. Using these estimates and based upon a total of 24 hospitals, recruiting 20 patients per hospital should achieve a power of at least 0.8 when the compliance proportion under the intervention is 0.20.

**Table 2:** **Sample size estimates for the trial**

**Number of eligible patients recruited per hospital (M)**	**Proportion meeting the primary outcome criteria under the intervention (** ***P*** **)**		
	**0.15**	**0.20**	**0.25**
10	0.487	0.707	0.845
15	0.547	0.777	0.907
20	0.590	0.809	0.921
25	0.606	0.832	0.937

### Randomisation

As explained above, we used deaneries as the unit of randomisation to minimise contamination between hospitals within the same deanery. We defined two blocks of deaneries, a block of six including hospitals (outside of the trial) where research teams were already actively involved in other ENCEPH UK studies and a block of six where there were no such ongoing studies (Figure 1). An independent statistician randomised equal numbers of clusters within each block to the intervention and routine arms, blinded to hospital identity.

### Statistical analysis

We will analyse the results in R (http://www.r-project.org) using a generalised linear mixed model [28] with binomial errors, logistic link, fixed effects for blocks and treatments, random effects for deanery and for hospital. If the intervention is effective, it will raise awareness of suspected encephalitis and lead to increased documentation in the clinical records of encephalitis as a differential diagnosis. In our baseline (pre-intervention analysis), we found that cases mainly included on this basis (method two) tended to be less likely to comply with the primary outcome criteria. One explanation is that these may represent clinical presentations at lower risk of having encephalitis and may therefore tend to be investigated and treated less thoroughly compared with more strongly suspected cases. It is therefore likely that the intervention will result in differential case mixes between the intervention and control arms, in this scenario diluting any real intervention effect. In our analysis of the outcome data, we will adjust for any relationship between the method of inclusion and compliance with the primary outcomes and analysis will be undertaken by a statistician blind to hospital assignment.

### Cost-effectiveness analysis

The economic evaluation will take the perspective of the UK National Health Service (NHS). We will use trial records and observations to estimate the costs of intervention delivery. Data on health care resource utilisation will be gathered from case record reviews. Unit costs will be obtained from publicly available routine data [29,30]. Costs and health benefits occurring over 12 months will be discounted at 3.5% per annum. Therefore, results will include, in addition to incremental costs and benefits, an estimate of total cost per patient for each arm. In order to estimate quality-adjusted life years (QALYs), utility values will be estimated from the results of administering the EQ-5D, primary economic outcome, and the SF-6D utility scores derived from the SF-36, following Brazier’s methodology [31].

We will analyse cost data using regression methods for handling censored cost data and accounting for clustered nature of the data [32]. We will analyse costs and benefits jointly using a bivariate probability distribution. Sample uncertainty in estimated cost difference and incremental cost-effectiveness ratios between arm groups will be described using bootstrapped confidence intervals [33] as well as with cost-effectiveness acceptability curves. Parameter uncertainty and robustness of findings will be accounted for through univariate sensitivity analysis and probabilistic sensitivity analysis.

Estimates of costs and health outcome (utilities) will be used to populate a model of suspected encephalitis patient management in both intervention and control conditions. The model will cover the remaining patient lifetime within which the (probability of) cost-effectiveness of the alternative will be analysed [34]. Data analysis will be performed using Stata [35].

### Ethical review

The study was reviewed by Preston North West Research Ethics Committee (13/NW/0279) (Additional file 2).

### Trial status

The trial is ongoing with pre-intervention data collection completed; we are yet to undertake post-intervention outcome data collection and analysis.

## Additional files

Additional file 1:
**CONSORT 2010 checklist of information to include when reporting a cluster randomised trial.**
Additional file 2:
**Confirmation of ethical approval.**
