# Supplementary material for: The evaluation of a tailored intervention to improve the management of suspected viral encephalitis: protocol for a cluster randomised controlled trial
Source: Implement Sci. 2015 Jan 27;10:14. doi: 10.1186/s13012-014-0201-1 (PMC4314797; doi:10.1186/s13012-014-0201-1)
Supplement: Additional file 2: — Confirmation of ethical approval. [file 13012_2014_201_MOESM2_ESM.pdf]

Health Research Authority
National Research Ethics Service

NRES Committee North West - Preston
HRA NRES Centre - Manchester
Barlow House
3rd Floor
4 Minshull Street
Manchester
M1 3DZ

Telephone: 0161 625 7818
Facsimile: 0161 625 7299

03 May 2013

Professor Tom Solomon
Chair of Neurology and MRC Senior Clinical Fellow
The University of Liverpool
Institute of Infection and Global Health, University of Liverpool
8 West Derby Street, Liverpool
L69 7BE

Dear Professor Solomon

**Study title:**	**Development and evaluation of an intervention based around the National guidelines on the management of suspected encephalitis, and its evaluation through a cost effectiveness analysis.**
**REC reference:**	**13/NW/0279**
**Protocol number:**	**UoL000915**
**IRAS project ID:**	**83084**

The Research Ethics Committee reviewed the above application at the meeting held on 26 April 2013. The Committee thanks Dr Kneen and Dr Backman for attending to discuss the application.

We plan to publish your research summary wording for the above study on the NRES website, together with your contact details, unless you expressly withhold permission to do so. Publication will be no earlier than three months from the date of this favourable opinion letter. Should you wish to provide a substitute contact point, require further information, or wish to withhold permission to publish, please contact the Co-ordinator Mrs Carol Ebenezer, nrescommittee.northwest-preston@nhs.net.

Ethical opinion

The Chair welcomed Dr Kneen and Dr Backman to the REC and thanked them for attending to discuss the application. The researchers gave apologies for Professor Solomon who had been unable to attend. The researchers agreed to the presence of the observer for the discussion and decision on their application.

The Committee told the researchers that this is a well put together and worthwhile project.

The Committee asked for clarification of the intervention. The researchers stated that it is difficult to explain as it is still in discovery phase. They plan to interview 40 health care

professionals and do a thematic analysis. They will then focus on the problems identified and the ideal characteristics of an intervention, e.g. it could be a teaching session. It will be a package comprising several components and will be standardised for use across the hospitals.

The researchers asked whether the Committee would be happy to approve the application at this stage or whether they would need to come back to the Committee with an amendment of the intervention package.

The researchers left the room.

Following discussion the Committee decided that an amendment would not be necessary and that a favourable opinion could be given. The researchers were invited back to the room and were told of the Committee's decision. The Committee advised that the decision would be confirmed in writing.

The members of the Committee present gave a favourable ethical opinion of the above research on the basis described in the application form, protocol and supporting documentation, subject to the conditions specified below.

Ethical review of research sites

NHS Sites

The favourable opinion applies to all NHS sites taking part in the study, subject to management permission being obtained from the NHS/HSC R&D office prior to the start of the study (see "Conditions of the favourable opinion" below).

Non NHS sites

The Committee has not yet been notified of the outcome of any site-specific assessment (SSA) for the non-NHS research site(s) taking part in this study. The favourable opinion does not therefore apply to any non-NHS site at present. I will write to you again as soon as one Research Ethics Committee has notified the outcome of a SSA. In the meantime no study procedures should be initiated at non-NHS sites.

Conditions of the favourable opinion

The favourable opinion is subject to the following conditions being met prior to the start of the study.

Management permission or approval must be obtained from each host organisation prior to the start of the study at the site concerned.

Management permission ("R&D approval") should be sought from all NHS organisations involved in the study in accordance with NHS research governance arrangements.

Guidance on applying for NHS permission for research is available in the Integrated Research Application System or at http://www.rdforum.nhs.uk.

Where a NHS organisation's role in the study is limited to identifying and referring potential participants to research sites ("participant identification centre"), guidance should be sought from the R&D office on the information it requires to give permission for this activity.

For non-NHS sites, site management permission should be obtained in accordance with the procedures of the relevant host organisation.

Sponsors are not required to notify the Committee of approvals from host organisations

It is responsibility of the sponsor to ensure that all the conditions are complied with before the start of the study or its initiation at a particular site (as applicable).

Approved documents

The documents reviewed and approved at the meeting were:

Document	*Version*	*Date*
Covering Letter		28 March 2013
Evidence of insurance or indemnity		02 August 2012
Investigator CV	Prof Tom Solomon	01 March 2011
Letter from Sponsor		25 March 2013
Other: Letter from Funder		28 September 2009
Protocol	2.0	26 March 2013
REC application	3.5	28 March 2013
Referees or other scientific critique report	Prof Kennedy	20 March 2013
Referees or other scientific critique report	Monty Silverdale	20 March 2013

Membership of the Committee

The members of the Ethics Committee who were present at the meeting are listed on the attached sheet.

Statement of compliance

The Committee is constituted in accordance with the Governance Arrangements for Research Ethics Committees and complies fully with the Standard Operating Procedures for Research Ethics Committees in the UK.

After ethical review

<u>Reporting requirements</u>

The attached document “After ethical review – guidance for researchers” gives detailed guidance on reporting requirements for studies with a favourable opinion, including:

- Notifying substantial amendments
- Adding new sites and investigators
- Notification of serious breaches of the protocol
- Progress and safety reports
- Notifying the end of the study

The NRES website also provides guidance on these topics, which is updated in the light of changes in reporting requirements or procedures.

<u>Feedback</u>

You are invited to give your view of the service that you have received from the National Research Ethics Service and the application procedure. If you wish to make your views known

please use the feedback form available on the website.

Further information is available at National Research Ethics Service website > After Review

**13/NW/0279**	**Please quote this number on all correspondence**

We are pleased to welcome researchers and R & D staff at our NRES committee members' training days – see details at http://www.hra.nhs.uk/hra-training/

With the Committee's best wishes for the success of this project.

Yours sincerely

pp.

Dr Patricia Wilkinson
Chair

Email: nrescommittee.northwest-preston@nhs.net

Enclosures: *List of names and professions of members who were present at the meeting and those who submitted written comments*
"After ethical review – guidance for researchers"

Copy to: *Mr Alex Astor*
Mr Mike Morris, The Walton Centre for Neurology and Neurosurgery NHS Trust

NRES Committee North West - Preston

Attendance at Committee meeting on 26 April 2013

Committee Members:

Name	*Profession*	*Present*	*Notes*
Mr David Abbotts	Lay member	Yes	
Dr Anoop Chauhan	Consultant Cardiologist	Yes	
Mr John Dalton	Lay Member	Yes	
Mrs Debbie Foord	Service Improvement Manager	Yes	
Mr Mike Hammond	Lay Member	No	
Mrs Kate Kilshaw	Radiographer	Yes	
Ms Joanna Marshall	Nurse Specialist in Acute Pain	Yes	
Dr Rob Monks	Senior Lecturer Department of Nursing	Yes	
Professor Videsh Raut	Consultant Orthopaedic Surgeon	Yes	
Mrs Vasanthi Vasudevan	Diabetes Research Nurse	Yes	
Dr Patricia Wilkinson	General Practitioner/ Chair	Yes	

Also in attendance:

Name	*Position (or reason for attending)*
Miss Anna Bannister	Assistant Coordinator
Jacqueline Bradley	Senior Paediatric Research Nurse Observer
Mrs Carol Ebenezer	Committee Co-ordinator
